# Water safe Worcester: student-led drowning prevention in an adolescent underserved population

**DOI:** 10.3389/fpubh.2024.1387094

**Published:** 2024-07-10

**Authors:** Katharine Playter, Erin Hurley, Kendall Lavin-Parsons, Kurren Parida, Zachary Ballinger, Kaitlyn Wong, Alycia Valente

**Affiliations:** ^1^UMass Chan Medical School, Worcester, MA, United States; ^2^Department of Pediatric Surgery, UMass Chan Medical School, Worcester, MA, United States; ^3^UMass Memorial Medical Center, Worcester, MA, United States; ^4^Department of Emergency Medicine, UMass Chan Medical School, Worcester, MA, United States

**Keywords:** drowning (prevention and control), adolescence, swimming, water safety, BIPOC

## Abstract

Adolescents aged 15 to 19 years have the second highest fatal drowning rate of any age group, second only to toddlers aged 12 to 36 months. This risk is amplified in black, indigenous, and people of color (BIPOC), and those of low socioeconomic status. Worcester, MA is a diverse city with over 40% of residents identifying as BIPOC and 20% living below the poverty line. The city has multiple natural bodies of water available for recreation, putting Worcester residents, particularly adolescents, at high risk of drowning. It is known that swimming lessons provided to adolescents significantly improve their swimming skills, however many programs are tailored to young children and are not appropriate for adolescents. Students from the University of Massachusetts T.H. Chan Medical School (UMass Chan), in collaboration with community partners, developed a water safety and swim education program tailored to Worcester adolescents as a means for an age-appropriate swim experience and education, community engagement, and injury prevention. Water Safe Worcester (WSW) was established as a city-wide injury prevention program that included swim lessons offered by medical students at the Central Community Branch YMCA in Worcester, MA. Instructors included UMass Chan medical students, graduate students, and staff. Adolescent YMCA members were invited to participate in lessons free of charge. Lessons were 90 min and emphasized a 3-fold approach: (1) expand knowledge of water safety and what to do in an emergency, (2) increase swimming skills, and (3) reduce fear of water. The overall attendance for the 2023 spring and summer sessions offered was 73 students, including multiple swimmers who attended more than one session. A total of 12 volunteers participated, which included 9 first-year medical students, one PhD student, one research assistant, and one surgery resident from UMass Chan. WSW demonstrated promising outcomes during its swim education classes, suggesting that WSW is a successful model to promote water safety, reduce the risk of drowning, and expand access to life-saving skills to Worcester's at-risk adolescents. This program serves as a critical step toward health equity while also providing an avenue for public health and injury prevention exposure for medical students.

## Introduction

Drowning is the third leading cause of unintentional injury-related death, and accounts for 7% of deaths worldwide (1). Increasingly, these deaths have been occurring in natural bodies of water as opposed to pools with lifeguards. Drowning rates have also been found to vary by age. Specifically within the pediatric population, toddlers aged 12 to 36 months have the highest fatal drowning rate, while adolescents aged 15-19 years have the second highest fatal drowning rate (1). Among the latter age group, individuals at greatest risk include those who are black, indigenous, people of color (BIPOC), male, and of lower socioeconomic status (1). Although overall drowning rates have been declining nationally since 2010, the increase in individuals recreating in natural bodies of water during the COVID-19 pandemic led to a 26% increase in drowning rates in such environments (2). Current estimates indicate that over 85% of all drowning cases could have been prevented through supervision, swimming instruction, regulation, and public education (3).

Swimming is a skill that saves lives, but it has historically been accessible along racial and socioeconomic lines. Systemic racism stemming from segregation has played an undeniable role in generations of racial disparity in swimming ability due to chronic lack of access to both public and private pools for BIPOC individuals (4). This inequity is also reflected in racial disparities regarding the fear of drowning, a concern strongly correlated with limited swimming skill; black women are more likely to report concerns of drowning than any other group (5). The cumulative effect of these systemic barriers is evident in a drowning rate that is 7.6 times higher among black children aged 10-14 and 2.5 times higher among black adolescents aged 15-19 compared to their white counterparts in the same age group (6). Worcester, Massachusetts is a diverse city with over 40% of Worcester residents identifying as BIPOC and 20% living below the poverty line, according to a 2021 census (7). The city's proximity to multiple natural bodies of water, as well as its at-risk population, creates a continuous need for water safety and swimming education in the greater Worcester area.

### Setting

#### Worcester, MA

Worcester's diversity, levels of poverty and significant refugee population make residents especially vulnerable to swimming-related death. According to the 2021 census, 12.7% of the city's population identifies as Black, 23.9% identifies as Latinx and over 22% report being foreign-born, with the latter accounting for 30% of the total refugee population in Massachusetts (7). Immigrants and refugees are at particularly high risk for drowning (8). An analysis of Massachusetts drownings from 1999 to 2013 revealed that immigrants were 42% more likely to drown than the general population, and the children of immigrants were 64% more likely to drown than children from non-immigrant families (9). This disparity is thought to be related to language barriers as well as a lack of familiarity for a new geographic, social, and cultural climate (10).

With five natural bodies of water near downtown Worcester serving as recreational swimming areas for residents, Worcester's natural landscape poses a drowning risk for residents, regardless of their swimming ability. Swimming in natural bodies of water presents a higher risk profile, as swimmers are more likely to encounter unpredictable conditions. These recreational areas also tend to lack lifeguard supervision and regulatory oversight (11).

This risk was brought into focus in 2021, when a local police officer, Emmanuel “Manny” Familia, died alongside a 14-year-old boy he was attempting to rescue during an incident at a local Worcester pond. Elements contributing to this tragedy included the adolescent victim's lack of prior swimming exposure, education, and knowledge of their drowning risk. Other factors include inadequate signage and public health intervention in the community, as well as lack of proper water rescue equipment for Worcester police officers. This event was a reminder of the need for increased water safety in Worcester and revealed the significant lack of injury prevention and education platforms for water-related injuries, particularly amongst minority adolescent community members. In the wake of this event, the Manny 267 Foundation was formed to honor Officer Familia's legacy with a similar mission to expand water safety in Worcester. The foundation has evolved into a crucial community partner for WSW, serving as a launchpad for the broader expansion of water safety initiatives in the community.

The need for comprehensive water safety measures in the Worcester community has never been more apparent. The vulnerability of Worcester's diverse population, coupled with the accessibility of natural bodies of water, underscores the urgency for targeted interventions to prevent further instances of drowning, leading to the establishment of WSW.

## Program overview

Swimming lessons are a well-established approach to drowning prevention, and previous intervention work has shown that formal swimming lessons among minority children and adolescents significantly improved their swimming skills (12–14). “Water Safe Worcester” (WSW) is a, multifaceted approach to drowning prevention created by medical students under the guidance of physician mentors at the University of Massachusetts T.H. Chan Medical School (UMass Chan). The program includes Hands-Only CPR instruction, public health messaging, on-land water safety materials, and partnership with community organizations to provide in-water education sessions. During a 3-week pilot program and 6-week summer program, WSW-affiliated students and staff led free swim lessons to an underserved adolescent population at the Central Community Branch YMCA in Worcester, MA. In addition to providing a needed community resource, WSW was also able to establish a unique avenue through which medical students were able to gain experience in public health and injury prevention, education opportunities, and build strong community connections. This paper will describe the preliminary approach that Water Safe Worcester has taken to increase drowning prevention efforts in the Worcester community, challenges faced in the process, and future goals the project.

### Program design

Private swim lessons are expensive and are often cost-prohibitive for those of low socioeconomic status (15). Worcester's YMCA and other publicly funded community programs offer swim lessons, which is an important first step in addressing the underlying disparity in swimming access. However, these lessons still cost between $60 and $100 for a 6-week course (16), and multiple courses are typically required to be considered a competent swimmer. The additional costs of swimsuits, goggles, and transportation all contribute to the significant barriers to learning to swim for many Worcester residents. WSW, in conjunction with its community partners, provided pool access, free instruction, and the necessary equipment to eliminate these common obstacles to swimming participation.

Adolescents attempting to learn to swim also face age-specific challenges. Typical beginner programs are designed for younger children, which may hinder adolescent participation in the lessons. Although the initial skill level between children and adolescents may be similar, their learning needs are distinct for their age and experience. Adolescents are stronger and more coordinated than younger children, allowing them to master skills more quickly (17). However, they have also had more time to establish a fear of the water and are more likely to have had adverse experiences related to swimming, requiring the instructor's specific attention and sensitive approach (18). Further, placing adolescents in lessons alongside elementary-aged children may discourage them from returning because of embarrassment for their skill level and lack of peer support. Instructors must be able to work with adolescents to tailor swim instruction to their unique psychosocial, physical and cultural needs.

#### Partnerships and funding

Community partnerships were a key to achieving WSW's goal to provide access to pool and swimming education and engage with the adolescent population of Worcester, MA. The primary community collaborator of WSW was the Central Community Branch YMCA, which provided a pool staffed with lifeguards for the lessons, volunteers to support and encourage adolescent participation, and helped foster general community interest for the events. The Manny 267 Foundation provided support for the program in helping to establish the initial connection and continuity with the YMCA and other teen-based programs as we launched this initiative and expanded its scope. Through these invaluable community partnerships, WSW has been able to offer adolescent-focused swim lessons at no cost and with no prior registration required. The program received community support from the city of Worcester and thus secured additional funding support from the District Attorney's Community Reinvestment and Crime Prevention Program.

### Concept and methods

Previous work on water safety education has highlighted the importance of peer-to-peer relationships, social pressures toward risk taking behavior, and normative comparisons to mentors/teachers as key factors in the effectiveness of water safety programs (19–22). Additionally, prior swim education programs have found that setting familiarity and comfort increases retention of material and emphasized that repeated exposure to safety teaching/resources was key to establish lasting change (23). As such, the WSW team chose to host the swim injury prevention and education program at the Central Community Branch YMCA in Worcester, MA. This branch was selected to provide geographic proximity to at-risk adolescents, easy access to previously established summer youth program personnel, and history of collaboration with outside institutions on long-lasting community efforts. The familiarity of the location, maintenance of pre-existing peer groups, and continuation of instruction from established YMCA faculty was a key in maximizing participant comfort and engagement. It also aided the formation and rapid strengthening of new relationships among the key stakeholders in the project, namely the participants, the Worcester community members, the medical professionals, and the program leadership.

WSW began as a pilot program, hosting free 90-min swim lessons weekly for 3 weeks at the YMCA in the Spring of 2023. UMass Chan affiliates, including medical students, PhD students, resident physicians, research staff, and other healthcare providers with any level of swim comfort were invited to volunteer as instructors. Prior to initiation of the pilot program, the Institutional Review Board (IRB) of UMass Chan reviewed this project and issued a Not Human Research approval. Any adolescent community member was welcome to participate. YMCA staff and teen leaders helped to garner interest and further participation among members by raising awareness of the program. Further, the team offered pizza and custom WSW merchandise to participating adolescents as an incentive for attendance. Following the success of the pilot program, the initiative was expanded into a 6-week program that ran through the summer of 2023. IRB approval was maintained for the expansion of the program.

Each WSW swim lesson was designed to provide education on injury prevention for both future encounters in the pool and in natural bodies of water outside of a pool setting according to recommendations outlined by the Water Safety USA organization (24). In accordance with these principals, the lessons emphasized a 3-fold approach: (1) expand knowledge of water safety and what to do in a water emergency, (2) increase swimming skills, and (3) reduce fear of water and drowning. In order to engage adolescents and maximize motivation, a variety of strategies were employed, including progressive task design, short instruction periods with frequent group rotation, and offering recognition for displays of acquired safety knowledge (24).

At the beginning of each session, swimmers were divided into groups based on self-reported skill level, swimming comfort, and social ties: friends were kept together in interest to promote camaraderie and mutual support during the learning experience. This resulted in an average student to teacher ratio of 2:1. Groups of swimmers would then rotate around the pool in 15-to-20-min increments through 3 to 4 stations of volunteers. Each station focused on a different topic, such as swimming skills like front and back floats, treading water, or stroke practice, or a water safety concept such as “Reach or Throw, Don't Go,” a water safety teaching strategy developed by the Red Cross that emphasizes extending an object to someone in distress, rather than entering the water (25).

Volunteer swim instructors from the medical community were not required to have experience as a swim instructor or lifeguard to work with WSW, however many people with that background were attracted to participate in the program. New and inexperienced volunteers were paired with individuals who had volunteered before and had experience teaching swimming lessons. Volunteers were instructed to tailor skill-based instruction to the level of the student. For example, first time participants in the program were taught the basics of breath control, floating, and front crawl swimming. More advanced students, however, engaged in activities that increased comfort and skill in the water, such as object retrieval and games that involved increased distances of swimming and increased breath control.

## Evaluation and effects

During the initial pilot program, an average of 11 students attended each WSW session at the YMCA, with the strongest presence being the program launch with 18 students attending. The summer program had an average attendance of seven students each session, with the strongest attendance on the final night of the program when 13 students attended. Since the initiation of the WSW swim program, a total of 73 student encounters were observed with several returning students throughout this program. A total of 12 UMass volunteers participated in the program, which included nine first-year medical students, one PhD student, one research assistant, and one surgery resident from UMass Chan Medical School (Table 1). Multiple instructors volunteered at multiple classes.

**Table 1:** Volunteer representation.

Medical students	12
PhD students	1
Residents	1
Research staff	1

Participants in the WSW swim lessons were asked to complete an informal, voluntary electronic survey before and after each lesson. The survey collected basic age distribution information and assessed individual swimming skills and attitudes toward water safety on a five-point Likert scale (Table 2). Challenges with survey collection in a pool setting resulted in a low response rate, and therefore quantitative conclusions were not drawn from results. Future survey investigation and optimizing ability to obtain higher compliance of these surveys in a pool setting will be evaluated to assess the perceived impact on swim safety and education on the adolescent student population.

**Table 2:** WSW survey questions.

*I am afraid to put my face in the water*
*I can float on my back for 1 min*
*When I am in the pool, I am afraid when the water is deep*
*I do not like to jump into the water*
*I feel confident that I can swim across the pool without stopping*
*I know what to do when someone near me is struggling in the water*
*I think that this session helped me feel more comfortable in the water*
*I want to come back again*

It has previously been observed that regardless of their swimming experience, adolescents, in particular teenage boys, tend to overestimate their abilities (26). Although many WSW participants had some experience with swimming, nearly all of the attendees lacked stamina. Approximately a third of participants were confident in their abilities to swim across the pool without stopping when asked, however volunteer instructors found this to be discordant with their observations. Moreover, many students claimed that they knew what to do in a water emergency, however, when the students were tested in an emergency scenario, nearly all lacked the knowledge of how to manage the situation. Instruction was tailored to individuals who overestimated their skills, emphasizing form and endurance, as well as overall safe water practices. Despite our original intent to prioritize adolescents with no swimming experience, we found that it was often those with some swim experience and overestimated their abilities, who may benefit the most from WSW encounters.

Alternatively, many swimmers approached classes with trepidation and fear of water, and some swimmers were hesitant to enter the water at all. Through encouragement and the employment of life vests, even apprehensive students were able to participate and practice safety skills in the pool. Informally assessing these students after class revealed a reduction in fear of water among these individuals and an overall increase in confidence compared to the start of class.

Hosting free swim lessons and injury prevention education classes for adolescents through the WSW initiative not only provided a service to the community, but also offered benefits to medical students involved. The program presented a unique opportunity for medical students to engage with adolescents in the community in a non-clinical setting. By participating in this initiative, medical students gained valuable hands-on experience in injury prevention and community medicine, further enhancing their education and understanding of public health issues.

## Discussion

This program sought to establish a targeted effort to address swimming proficiency disparities among BIPOC individuals within the Worcester community. Previous work has consistently demonstrated that involvement in swimming and water safety programs results in significant improvement in swimming ability and safety skills among children and young adults, including BIPOC populations (12, 13, 27). The present study found that providing free swimming lessons at a central community location was an effective avenue for enabling participation in swimming lessons and water safety education among the target population.

Notable trends were observed among participation and enrollment among Water Safe Worcester adolescents. Many participants had some prior swimming exposure; we approximated that over half of WSW participants had at least some prior swimming experience. Prior literature has shown, however, that previous participation in formal swimming lessons or swimming within a school curriculum had no significant impact on baseline water safety knowledge or attitudes (27). This suggests that baseline exposure to the water does not correlate with water safety skill, underscoring the importance of a multi-strategy approach toward water safety skill acquisition to specifically target drowning reduction.

Enlistment and retention of those without any water exposure was limited. This is likely related to a selection bias in the community, as those who attend the YMCA have the advantage and support of the community center, as well as access to its pool. This may also be related to a participant's inherent inclination for activity. It has been observed that there is a positive correlation between children's self-reported attitudes toward activity, and their self-reported and actual ability to swim (28).

A gender disparity was also noted among participants, with teenage boys comprising the majority of participants. Increasing participation among teenage girls, particularly those who identify as BIPOC, is essential because research indicates that they are the population that has the highest self-reported fear of drowning and are more likely to self-report not knowing how to swim than males (5, 29). To combat this disparity, the organization emphasized an inclusive atmosphere at each event and worked to recruit instructors of all genders.

Attendance and retention among program participants were crucial factors to achieving the program objectives and consistent challenges throughout the length the program. Adolescents' motivation to attend youth programs can significantly impact program outcomes. It has been found that while program content initially drives attendance of youth programs, the quality of relationships with staff and peers is the primary factor guiding retention (30). In this program, similar trends were observed; participants who engaged positively with volunteers were more likely to return to the program. Creating a supportive and inclusive environment where participants feel valued can enhance attendance and retention rates, particularly in those who face barriers to participation.

### Limitations

Collecting survey responses among an adolescent population on a pool deck presented a difficulty; neither paper nor electronic surveys were ideal for a pool environment, and adolescent participants exhibited limited patience for survey completion. It was difficult to incentivize the participants to stay after the lesson to complete the post-survey and unrealistic to expect adolescents on the pool deck to thoughtfully respond to lengthy survey questions without incentivization. Compliance with surveys remained a significant barrier to the investigation of the student evaluation of WSW. This warrants an investigation into the efficacy of the survey designed for the purpose of eliciting responses from an adolescent audience. Considering survey wording and length, closed format questions, and minimizing negative wording is likely to enhance response rate and yield higher quality responses (31).

## Conclusion

Implementing free, community-based drowning interventions to promote basic water safety and swimming skills among racial and ethnic minorities is a necessary step in promoting equity and minimizing morbidity and mortality secondary to swim-related injuries. In establishing community relationships and providing practical swim education and water safety skills to adolescents, WSW demonstrated encouraging progress toward its goal during its inaugural summer of swim education classes. This experience suggests that the swimming portion of the WSW model holds promise as a sustainable way to promote water safety, reduce risk of drowning, and expand access to life saving skills to Worcester's at-risk residents, serving as a critical step toward health equity in the community. This work also affords medical students the opportunity to engage meaningfully with their community and gain valuable hands-on experience in injury prevention and community medicine. The popularity of the swimming portion of this program builds a trustworthy foundation to increase community buy-in for WSW's comprehensive, multi-strategy water safety program. The success of the WSW program underscores the importance and effectiveness of community-based drowning initiatives in promoting health equity and minimizing swim-related injuries among racial and ethnic minorities.

### Take home points

1. Free, accessible swim instruction confronts racial disparities in swimming proficiency, bridges gaps, and promotes water safety among those at highest risk for drowning by tailoring lessons toward adolescents from underserved communities.
2. Providing instruction in a location that is familiar and convenient ensures that participants can easily access the program while building their trust with medical students that can extend to the broader medical community.
3. Medical student-led volunteer initiatives create a unique opportunity for local adolescents to build relationships with medical students, creating a supportive, peer-led environment where adolescents feel comfortable learning water safety skills and asking questions.
4. Swim lessons play an important role in building confidence and reducing the fear of drowning among participating adolescents.
5. WSW serves as a framework for medical student-led community engagement and public health intervention.

## Data availability statement

The original contributions presented in the study are included in the article/supplementary material, further inquiries can be directed to the corresponding author.

## Author contributions

KPl: Conceptualization, Formal analysis, Investigation, Methodology, Project administration, Writing – original draft, Writing – review & editing. EH: Conceptualization, Formal analysis, Investigation, Methodology, Project administration, Writing – original draft, Writing – review & editing. KL-P: Conceptualization, Formal analysis, Investigation, Methodology, Project administration, Writing – original draft, Writing – review & editing. KPa: Conceptualization, Methodology, Project administration, Visualization, Writing – original draft, Writing – review & editing. ZB: Conceptualization, Methodology, Project administration, Supervision, Writing – original draft, Writing – review & editing. KW: Conceptualization, Formal analysis, Investigation, Project administration, Resources, Supervision, Visualization, Writing – original draft, Writing – review & editing. AV: Conceptualization, Formal analysis, Investigation, Methodology, Project administration, Resources, Supervision, Visualization, Writing – original draft, Writing – review & editing.
